# Designing digital health interventions with causal inference and multi-armed bandits: a review

**DOI:** 10.3389/fdgth.2025.1435917

**Published:** 2025-06-05

**Authors:** Radoslava Švihrová, Alvise Dei Rossi, Davide Marzorati, Athina Tzovara, Francesca Dalia Faraci

**Affiliations:** ^1^ Institute of Computer Science, Faculty of Science, University of Bern, Bern, Switzerland; ^2^ Institute of Digital Technologies for Personalized Healthcare, Department of Innovative Technologies, University of Applied Sciences and Arts of Southern Switzerland, Lugano, Switzerland; ^3^ Faculty of Informatics, Universitá Della Svizzera Italiana, Lugano, Switzerland; ^4^ Center for Experimental Neurology, Department of Neurology, Inselspital, Bern University Hospital and University of Bern, Bern, Switzerland

**Keywords:** behavioral change, causal inference, digital health, interventions, micro-randomized trials, multi-armed bandit, personalized medicine, statistical analysis

## Abstract

Recent statistics from the World Health Organization show that non-communicable diseases account for 74% of global fatalities, with lifestyle playing a pivotal role in their development. Promoting healthier behaviors and targeting modifiable risk factors can significantly improve both life expectancy and quality of life. The widespread adoption of smartphones and wearable devices enables continuous, in-the-wild monitoring of daily habits, opening new opportunities for personalized, data-driven health interventions. This paper provides an overview of the advancements, challenges, and future directions in translating principles of lifestyle medicine and behavior change into AI-powered mobile health (mHealth) applications, with a focus on Just-In-Time Adaptive Interventions. Considerations for the design of adaptive interventions that leverage wearable and contextual data to dynamically personalize behavioral change strategies in real time are discussed. Bayesian multi-armed bandits from reinforcement learning are exploited as a framework for tailoring interventions, with causal inference methods used to incorporate structural assumptions about the user’s behavior. Furthermore, strategies for evaluation at both individual and population levels are presented, with causal inference tools to further guide unbiased estimates. A running example of a simple real-world scenario aimed at increasing physical activity through digital interventions is used throughout the paper. With input from domain experts, the proposed approach is generalizable to a wide range of behavior change use cases.

## 1 Introduction

Non-communicable diseases (NCDs) are a challenge to global public health (1). WHO statistics show that NCDs account for a substantial majority (74%) of worldwide mortality (1). Among the leading contributors to this burden are cancer, diabetes, and cardiovascular diseases, each linked to lifestyle factors such as diet, physical activity, and tobacco use (2). In recent years, there has been a growing recognition of the potential for lifestyle interventions to mitigate the prevalence of NCDs and improve overall health outcomes (3).

With the global wide adoption of mobile and wearable devices and associated applications, lifestyle management through health apps is becoming more and more accessible to a broader population (4). Emerging technologies offer novel opportunities for real-time monitoring, personalized feedback, and intervention delivery, thereby empowering individuals to take charge of their health in modern ways and in clinical practice (5, 6). In parallel, the fast raising field of artificial intelligence (AI) holds extensive promise for enhancing the effectiveness and scalability of behavior change interventions (7–9). By harnessing the power of machine learning algorithms, AI can analyze vast amounts of data to derive insights from individual behaviors, preferences, and contexts, thus enabling the delivery of tailored interventions that resonate with users on a personal level (10).

The present work reviews the current perspectives and challenges in both designing and evaluating the mHealth behavior change solutions. After introducing the concepts of lifestyle medicine and behavioral change, we address how to integrate them in behavioral change support systems that leverage personalization techniques, including just-in-time adaptive interventions. Further, usage of bayesian multi-armed bandits to enhance user engagement and adherence to lifestyle change programs is discussed. In the second part of the paper, current evaluation techniques for interventional data are presented, with focus on sequential designs. Finally, recent challenges and potential developments in the field are described. Throughout this paper, we will consider as a use case scenario the design of a digital intervention system aimed at improving the level of *physical activity*, which represents one of the fundamental lifestyle pillars. The discussed framework is however generalizable, after input from experts in the field, to any of the other lifestyle pillars or their combination.

### 1.1 Lifestyle medicine

Incorporating principles of lifestyle medicine into a mobile app for interventions is essential for promoting holistic health and well-being. Lifestyle medicine is a branch of evidence-based medicine focusing on comprehensive behavioral changes through interventions aiming to prevent, manage, and sometimes even treat various health conditions (11). The role of lifestyle factors in influencing health outcomes is emphasized. According to (12) there are 6 pillars of lifestyle medicine recognizing impact on overall health. These are namely: mental well-being and stress-management; healthy relationships; healthy eating; physical activity; sleep; minimizing harmful substances.

The aim of lifestyle medicine is to address the root causes of health issues by promoting healthy behaviors and habits (11). Lifestyle medicine is often integrated into conventional medical practices and may involve collaboration among healthcare professionals, including physicians, dietitians, exercise physiologists, psychologists, and other specialists. It’s a holistic approach that considers the interconnectedness of various lifestyle factors and their impact on health.

#### 1.1.1 Behavioral change

Comprehending the basics of psychology related to behavior change is imperative when creating an app for an intervention framework (13). According to the transtheoretical model of health behavior change (14) there are 6 stages of behavioral change: precontemplation, contemplation, preparation, action, maintenance and termination. In precontemplation stage a need of change is formulated, followed by contemplation stage with motivation and definition of distal (long-term) goals and plans. During the preparation stage, the individual sets realistic proximal (short-term) goals and gets ready to take action. In the action phase interventions are followed aiming to fulfill proximal goals. After 6 months of actions the maintainance phase is reached, habit is created and should be maintained until termination stage, when user has no desire to return to old behaviors. Interventions related to different stages require different strategies and design. In this paper we will focus on discussing the development and design of digital interventions for action phase, i.e., the formation of a new habit aiming for getting to maintenance and hopefully termination stage (15). Interventions could be further supported by educational and motivational elements to enhance adherence. Further considerations for development of mHealth interventions can be found in (16).

#### 1.1.2 Goal setting and habit formation

One promising way to lead individuals towards behavioral change and habit formation is through goal setting strategies. Distal goals (long-term) are achieved through a set of proximal (short-term) goals (17) that can develop into tiny habits (18). Distal goals are typically used for action planning and are formulated to enhance intrinsic motivation (19), whereas proximal goals are formulated more specifically to guide daily behavior. For proximal goal setting, the SMART (specific, measurable, achievable, relevant, and time-bound) framework can be used (20). A plan to achieve the distal goal thus consists of a hierarchical set of executable proximal goals that, when performed regularly, foster long-term lifestyle changes (21).

For example, in the context of a digital intervention aimed at improving physical activity, a distal goal could be stated as “*Be physically more active*,” while a corresponding SMART proximal goal could be: “*I will walk 7,000 steps at least 5 days in a week for 4 months, to increase my physical activity*.” This goal is specific about the action, measurable and time-bound. The relevance and achievability however, are subjective and must be tailored to the individual context and capabilities. When applying mHealth solutions in clinical practice, incremental sub-goals should be consulted with clinicians and aligned with the patient’s medical profile. Additional aspects for designing effective SMART goals are addressed by (22).

Along with structural goal-setting, the psychological mechanisms behind motivation and habit formation should also be considered for digital health interventions design (23). Formation of new healthy habits, simultaneous to breaking existing unhealthy ones (24), is facilitated through repeated behavior in stable contexts (25, 26). This is often described as cue-routine-reward loops (27). Digital health interventions can reinforce these loops by providing contextual cues and reinforcing feedback. Daily actions prompted by digital interventions can be complemented by reflective processes occurring over longer behavioral episodes, allowing for temporal alignment with real-world behaviors and capturing the dynamic interplay between behavioral and cognitive constructs (28). Moreover, theories of motivation, particularly the distinction between intrinsic and extrinsic motivation, play a pivotal role in long-term adherence. While extrinsic motivators such as reminders or rewards may initiate a behavior, the transition toward intrinsic motivation (e.g., walking because it feels good or aligns with one’s values) is crucial for sustainability (29). Self-determination theory further suggests that behaviors are more likely to be maintained when they support a sense of autonomy, competence, and relatedness (30, 31). In mHealth applications, fostering *autonomy* can be facilitated through offering personalized goal setting and giving users choices in how they engage with interventions (e.g., selecting preferred types of activity or timing of reminders). *Competence* can be enhanced through clear feedback in form of e.g., progress tracking, with positive reinforcement features like daily streaks or milestone badges. To support *relatedness*, social features such as peer support groups, sharing achievements, or connecting with coaches and clinicians can help users feel part of a community. Additionally, adaptive interventions that evolve with the user’s behavior and needs over time can further enhance engagement. By aligning design elements with psychological principles, mHealth tools can move beyond compliance to enable meaningful, sustained behavior change.

## 2 Methods

In this section, we outline key methodological concepts: the causal inference framework for unbiased effect estimation and multi-armed bandit algorithms for adaptive decision-making. These concepts provide the foundation for the design of intervention components and evaluation strategies discussed in the following sections.

### 2.1 Causal inference

Many questions in health and behavioral sciences are inherently causal rather than purely associative (32). The aim of causal inference is to move beyond mere associations between variables, towards understanding and quantifying the causal effect of an exposure on an outcome. It distinguishes correlation from causation by relying on formal assumptions, conceptual frameworks, and methods that allow for the estimation of causal effects even in the presence of confounding, selection bias, and measurement error (32). Moreover, unlike purely predictive models, causal methods also seek to answer *counterfactual* questions: What would have happened to an individual or a population if a different action had been taken?

A foundational perspective in causal inference is the potential outcomes framework, also known as the Neyman-Rubin model (33). In this approach, each unit (e.g., an individual) is considered to have multiple potential outcomes, one for each possible treatment condition. For instance, a patient might have one outcome if treated with a drug and another if given a placebo. The causal effect is defined as a contrast between these potential outcomes, such as their difference or ratio. However, only one outcome is observed for each unit (the factual outcome), while the others remain unobserved (counterfactuals) (34). This constitutes the fundamental problem of causal inference: the impossibility of observing all potential outcomes for a single unit.

To make causal statements despite this limitation, researchers must rely on *assumptions* and designs that allow *identification* of causal effects. Central to this are the notions of *treatment* (or exposure), *outcome*, and *confounders* (variables that influence both treatment and outcome). Confounders can create spurious associations if not properly controlled, making their identification and *adjustment* essential for unbiased causal effect estimation (32).

Complementary to the potential outcomes approach is the use of graphical models representing causal relationships between variables. These graphs encode assumptions about the underlying data-generating process and enable formal reasoning about causation through graphical criteria such as d-separation and backdoor paths (32). When the structure of the graph is known or estimated, these tools provide a basis for identifying causal effects and designing appropriate adjustment strategies. A framework unifying the two paradigms, called single world intervention graphs (SWIGs), was presented in (35).

#### 2.1.1 Structural causal model

A central development in causal inference is the formulation of structural causal models (SCMs). SCMs formalize causal assumptions through structural equations that describe how each variable is generated from its causes and an exogenous error term (36). The relationships among variables can be represented graphically as a directed acyclic graph (DAG), where nodes correspond to variables and directed edges represent direct causal influences. The DAG not only captures causal mechanisms but also encodes conditional independence relationships implied by the underlying causal structure. Understanding the graph structure is fundamental, as it dictates how we can adjust for confounding, predict the effects of interventions, and identify causal effects from data.

##### 2.1.1.1 Causal discovery

In many applications, the true causal graph is unknown and must be inferred from observational or interventional data. Causal discovery, also known as structure learning, attempts to learn the structure of the underlying DAG, by estimating its equivalence class. Constraint-based methods, like the Peter-Clark (37) or Fact Causal Inference (38), infer graph structures by systematically testing conditional independencies, while score-based methods search for the graph that best fits the data according to a predefined scoring criterion. Whenever possible, a background knowledge should be incorporated, to restrict the search space and enhance causal discovery algorithm. If the variables are collected over multiple time points, causal discovery can be guided by known time orderings (39). Optimization-based methods, such as NOTEARS (40), on the other hand, employ gradient-based techniques, but currently lack built-in support for incorporating background knowledge. An overview of causal discovery methods can be found in (41). The output of causal discovery provides a foundation for subsequent causal analysis and inference.

#### 2.1.2 Effects and inference

With the potential outcomes and SCM frameworks in hand, we now focus on the task of estimating causal effects from the data. First, the DAG is used for identification of valid adjustment set via the back-door criterion, i.e., the smallest collection of covariates that blocks all noncausal paths from treatment to outcome (32). This step ensures that, under the assumptions of no unmeasured confounding and positivity, the target causal estimand (such as the average treatment effect) is identifiable (32).

Once the adjustment set is determined, the causal effect can be estimated through methods such as regression adjustment, where the outcome is modeled as a function of treatment and controls (confounders), and the predicted outcomes under different treatment levels are compared (42). The effect sizes of control variables, however, are unlikely to have causal interpretation themselves (43). Alternatively, inverse-probability weighting (IPW) (44) can be used, where each unit is weighted by the inverse of its probability of receiving the observed treatment, creating a pseudo-population in which treatment is independent of measured confounders. Another approach is the use of doubly robust methods (45–47), which combine outcome modeling with IPW to provide protection against misspecification in either of the models. Also meta-learners (48) utilizing machine learning techniques to estimate causal effect can be used. To estimate all effects on the graph, a (causal) Bayesian network (49) approach can be employed. In settings where the DAG is unavailable or incomplete, the intervention calculus when DAG is absent (IDA) method (50) can be used. This approach estimates the lower bound of total causal effects from observational data in high-dimensional settings, even without a fully specified causal graph. Additionally, by employing causal fusion, we can integrate data from both observational and experimental settings, as well as from multiple sources, enabling more robust causal inference across diverse datasets (51).

Finally, to assess the robustness of the derived findings to violations of unconfoundedness and to partially identify effects when point identification fails, sensitivity analysis can be performend and bounds estimated. The aim of sensitivity analysis is to quantify how strong an unmeasured confounder would have to be to overturn derived conclusions, using approaches such as Rosenbaum’s bounds (52) or the E-value (53). This allows for reporting, for example, the minimum strength of association that an unobserved variable must have with both treatment and outcome to explain away the estimated effect. When unmeasured confounding cannot be ruled out and point identification is questionable, bounds on the average causal effect can be estimated, e.g., via framework proposed in (54), and further advancements discussed in (55). Together, these sensitivity and bounding analyses complement the point estimates obtained under unconfoundedness and enhance the credibility of causal conclusions.

#### 2.1.3 Longitudinal setting

Estimating individual treatment effect from longitudinal data, i.e., repeated measurements, can be done by utilizing described concepts. In digital health, an N-of-1 analysis of self-tracked data (56) can be employed to evaluate causal effects in longitudinal observational data, by utilizing the counterfactual framework. Findings from individual N-of-1 analyses can then be combined to provide population-level treatment effect estimates (57). The main assumptions for these methods include the consistency of exposure assignments over time, no interference between individuals, and a proper specification of the counterfactual model. Another approach for evaluation of causal effects in time series data is Granger causality (58), which can be extended to non-linear effect evaluation with copulas (59). Furthermore, if the structure is unknown, several data-driven approaches, utilizing variants of constraint and score-based causal discovery methods, were proposed (60–62).

### 2.2 Multi-armed bandit

The multi-armed bandit (MAB) problem is a framework from reinforcement learning (RL), designed for *sequential decision-making* under uncertainty (63). In each time step t≥0, an *agent* with K arms (options) selects one arm k∈{1,…,K} according to a *policy*, and performs an action $a_{t}=k$. A *policy* is the agent’s strategy for selecting actions based on the information available up to time t. It can be a deterministic or stochastic rule that maps the agent’s current knowledge, typically the history of past actions and received rewards, into a choice of the next action. In the next time step t+1, as a consequence of its action, the agent receives a numerical reward $r_{t+1}$, and updates the estimate of an expected reward for that action, which is assumed to be *stationary*. The agent’s goal in MAB setting is to maximize expected total reward (63). Moreover, *regret*, defined as the difference between the reward that would have been obtained by always selecting the best arm and the reward actually accumulated, quantifies the cost of learning under uncertainty (64).

The true reward distributions associated with each arm are initially unknown and must be estimated through interaction with the environment (65). This introduces the exploration-exploitation trade-off: the agent must decide between *exploring* less certain arms to gather more information about their rewards and *exploiting* the arm that currently appears most promising. Several algorithms have been proposed to address the exploration-exploitation dilemma. Greedy algorithms prioritize the action with the highest estimated reward, favoring exploitation, whereas non-greedy strategies allow for occasional exploration to prevent premature convergence to suboptimal arms (63). Different MAB algorithms balance exploration and exploitation in various ways, with each approach yielding distinct theoretical and empirical properties (66). The process is memoryless, meaning the agent’s decisions at each time step depend only on the current policy, simplifying the analysis and implementation of learning algorithms.

#### 2.2.1 Contextual and causal MAB

To improve the performance of MAB algorithms, contextual information can be incorporated both in the action selection process and within the learning phase, such as informing the reward design (67). In this scenario, the policy is influenced not only by the agent’s previous actions but also by the *state* $s_{t}$ of the environment, which is assumed to be stationary in MAB setting.

In RL, action selection is based on the current state and policy (63). By introducing structural assumptions about the environment through SCM and an associated causal diagram representing actions of agent, this task evolves into causal RL (68). This can be reduced to causal MAB (69), which can exploit assumed SCM for better action selection if all variables are manipulatable. Moreover, an extension to handle also cases when not all variables are manipulatable was introduced in (70). This approach enables evaluation of how a decision-maker should intervene to optimize non manipulatable outcome which is causally connected to manipulatable variables.

#### 2.2.2 Dynamic environments

RL algorithms, including MABs, assume stationarity, i.e., the environment and reward distributions remain stable over time. This assumption however typically does not hold in dynamic environments, such as behavioral change programs. To tackle this, most recent observations can be treated as more important, by (possibly non linearly) weighting them more than past observations. For instance, this can be done with a sliding window approach, by setting the weight of past observations too far in the future to 0, effectively ignoring them, while only considering most recent ones.

A key challenge that remains open is the selection of an appropriate window size for adaptation, effectively a critical hyperparameter of the employed algorithm. The window size directly affects the trade-off between responsiveness to recent changes and robustness to noise. A small window can enable fast adaptation to sudden behavioral changes (e.g., illness or routine disruptions), but may result in instability due to overfitting to short-term fluctuations. Moreover, shorter window would lead to higher uncertainty (higher variance) in posterior distributions due to lower sample size, prioritizing exploration over exploitation in sampling. Conversely, a large window offers more stable estimates but may obscure important shifts in behavior. In practice, choosing the optimal window size depends on the expected rate of behavioral change and the variability of user engagement. A possible strategy to handle this could be fixing it to one plausible size according to previous studies or expertise. Another option would be selection of the window size in a data-driven way by discounted history weighting, or by exploiting models that can utilize past observations to modify the window size in response to shift in reward distribution (71, 72). An empirical comparison of possible strategies should be done to better tailor digital health solutions to the target population. Strategy of the window size adaptation is an important aspect to consider, as it directly impacts the algorithm’s ability to personalize interventions effectively over time. In the clinical and behavioral setting, window size choice could also take into consideration the need to reflect domain-specific rhythms, such as circadian cycles or weekly routines, to better capture meaningful patterns in individual’s behavior.

#### 2.2.3 Thompson sampling

Thompson sampling (TS) (73), a type of MAB, is a Bayesian approach to sequential decision-making under uncertainty, addressing the exploration—exploitation trade-off (72, 74). This algorithm operates by recomputing the posterior distribution after each received reward and thus building an implicit profile reflecting user’s preferences. Thanks to these two properties it is a reasonable choice for applications in designing of digital health interventions.

In TS, reward likelihood for each arm is represented by an unknown probability distribution. After performing an action by the selected arm and observing the associated reward, the posterior distribution over this arm is updated. To facilitate easier posterior updates, it’s advantageous to choose a conjugate prior distribution (75). If no information is available at the beginning, an uninformative prior should be chosen to start with, otherwise available data can be used to better initialize it. In case the reward of the k-th arm is binary, it can be described by a Bernoulli distribution with probability of success $p_{k}$, which can be modeled by Beta distribution $Be(\alpha_{k},\beta_{k})$ (76). In this case, for posterior update a Beta-Bernoulli is a convenient choice, as Beta distribution is a conjugate prior (75). For an uninformative prior, a Beta distribution with both $\alpha_{k},\beta_{k}$ equal to 1, i.e., a uniform distribution on support ⟨0,1⟩, can be used. For the outcome of count data (e.g., Poisson) and with possibly high frequency of zeroes, count and zero-inflated models can be incorporated (77).

The action selection process in TS involves sampling from the posterior distributions over arms and selecting the arm with the highest sampled probability of success. If the environment is not stationary, a sliding-window TS (78) algorithm can be used. Posterior distributions are then computed only from the M most recent actions and the associated rewards observed, assuming stationarity in this window. The window size can be dynamically selected, as discussed in Section 2.2.2. Moreover, updating posterior estimates in TS in batches (79) instead of daily could help maintain more consistent estimates of reward distributions across arms, albeit at the expense of higher variance.

If distributions over arms for TS in each step depend also on the contextual variables, the contextual TS (80) can be used. Furthermore, relevant variables used for modeling user’s state can be transformed to latent variables before applying it to TS (81), using statistical or machine learning models. If unobserved confounders are present, an adaptation of TS proposed in (82) can be employed. Another variant of TS capable of handling online environments was proposed in (83). Ultimately, the choice of the specific TS variant, or other MAB, is task-dependent and should be guided by both technical feasibility and specific requirements of the application.

## 3 Personalized adaptive interventions

In mHealth applications, a personalized *nudge* is a brief persuasive intervention, that encourages and motivates users towards a specific behavior (84). Intervention aiming for behavioral change can be delivered in a text form through push notifications, in-app messages or through conversational interfaces. Also haptic feedback (85) can be used, by signalizing e.g., milestones or providing real-time feedback as done in (86). Moreover, providing interim study results to participants (87) could enhance engagement. Other approaches not involving text itself that can serve as an intervention can be within a serious game, either via self-monitoring (88), rewards or other designs (89), or in other ways depending mainly on technical availabilities. The aim of intervention has to be clear, and motivational and persuasive techniques can be employed to enhance its efficacy. In this section, the opportunities and challenges in the design of digital interventions, based on the various data sources in mHealth applications, are discussed. To enhance the efficacy of digital interventions and user engagement, multi-armed bandits, introduced in Section 2.2, can be involved. MAB is preferred over RL thanks to it’s ability to adapt faster in the settings with little user interaction data in the early stages, prioritizing user retention through fast optimization. By focusing only on immediate feedback and avoiding the complexity of modeling long-term behavior, MAB allows for quicker adaptation and leads to lower regret compared to full RL.

### 3.1 Data sources

Collected data can be divided into three categories based on their source and characteristics: objective, subjective and contextual. Objective data are those that can be automatically collected through wearable devices such as smartwatches and smart rings (90). Examples of objective data collected from wearable devices are, just to name a few: steps, resting heart rate, heart rate variability, bedtime and wake up time, and physical activities. Objective data can also be collected through smartphones and mHealth app usage metrics (total time spent using an mHealth app, the way in which the app is used,…). Subjective data are, on the other hand, collected directly from the user, either through questionnaires, conversational interfaces, or digital diaries. Subjective data can then be used to derive *explicit* contextual information and to better understand objective measures. Furthermore, *implicit* contextual information, such as weather and date-related features (day of the week, season, …), are derived from smartphone background data (91). For our example, data about physical activity can be obtained either as number of steps per day, or as activity logs (type, distance, other metrics related to the activity) or as intensity minutes (time spent in heart rate intervals). Daily steps will be used for evaluation of proximal goal, and other metrics will be relevant for evaluation of improvement in distal goal. Most wearables can provide this type of information, but it can be collected also by subjective measures, although with higher uncertainty.

#### 3.1.1 Missing data

In the mHealth applications, missing and noisy data are a common problem. Non-availability of some data sources can be present, either due to users’ lack of technology adoption, missing resources (wearable device not used or data collected only passively with wearables but not using other mHealth app components) or because of regulatory or technical restrictions (92, 93). The behavioral change system must be then designed to be scalable and usable for everyone even with a minimal data flow, while ensuring availability of relevant data sources. This structure entails a range of components within the system, from establishing minimal data for the basic version to leveraging all available data sources for more advanced features. Such design ensures scalability, accommodating varying degrees of user system adoption (94).

Even if the adoption of all features was successful, gaps and noise in longitudinal data will persist. These gaps can arise from factors such as non-compliance, where patients fail to adhere to wearable device usage or mobile app protocols, technical issues with the devices themselves or simply during charging the device. To deal with non-compliance, various approaches can be used, involving design considerations to increase adherence, e.g., with gamification or reward system (95, 96), or financial incentives (97). Further, to encourage adherence in digital diaries, notifications sent at random or scheduled times (98) or based on the contextual information about user’s current state (99), if available, can be incorporated.

Handling missing data presents a critical challenge for the functioning of online behavioral change algorithms, particularly in digital health interventions. These “data holes” can disrupt real-time decision-making and compromise personalization and efficacy of interventions. Authors of (100) proposed a framework utilizing auxiliary variables to overcome this issue for handling missing accelerometer data in the outcome variables in trials, which could be adapted to other variables as needed. Further, in (101), expectation-maximization approach is compared to multiple imputation technique for missing step count data. To manage noisy data from wearables, signal processing approaches, such as filtering or denoising can be also utilized. If historical data about the user is available, imputation by utilizing similar context from previous days could be an option, however missingness mechanism might not be at random and this could lead to incorrect conclusions. Importantly, any behavioral decision based on incomplete data may be biased, and prolonged missingness can render the system non-functional. Therefore, ensuring data continuity, by the combination of technical means and fostering user adherence, remains a key operational priority in the design of robust digital interventions.

For the post-analysis, i.e., evaluation of distal goals, and when deriving clinical trial endpoints, statistical approaches for missing data including within-patient imputations across common time periods, functional data analysis, and deep learning methods can be used, as presented in (102). For longitudinal studies with dropouts or truncation, the approach utilizing inverse-probability weighting and generalized estimating equations proposed by (103) can be considered. Other considerations for prevention and treatment of missing data in clinical trials are summarized in (104).

### 3.2 Just-in-time adaptive interventions

The most important aspect of digital health interventions is *when* they are delivered, to aim for the optimal outcome, i.e., behavioral response. Multiple *decision points* can be thus defined, either during the day, week or other relevant cycle, that is targeted through the definition of proximal goals. These do not have to be fixed, subject to technical availabilities they can be also event-based, if online data are available. That is however unrealistic in most of the mHealth applications, and this work will focus on the selection from the set of decision points. The simplest timing modality is at a reasonable fixed time, or a random selection from multiple options. This is especially useful for new users, when no relevant information is available for the decision. If available, contextual information should be used to better decide if to deliver the intervention at each decision point.

To increase efficacy of the intervention, minimize disruption to daily life and avoid frustration or disengagement, user’s *internal state* should be determined prior to sending it (105). Nahum-Shani et al. (105) defines states of *vulnerability/opportunity* that aims to find a period of susceptibility to negative/positive health behavior changes, and *receptivity* targeting individual’s willingness to receive the support delivered with the intervention. These concepts are explained in more detail also in (99). For instance, a reminder to walk more, as in our example, while driving should be avoided, as attempted in (106). Such intervention would be not only disengaging, but could also have a negative impact on the trustworthiness of the system’s decisions.

The Just-In-Time Adaptive Interventions (JITAIs) framework, introduced by (99), addresses this challenge by tailoring intervention timing to a user’s current internal and contextual state. The decision rules are driven by defined thresholds for these *tailoring variables* and determine not only whether to intervene, but also the *type of intervention* to be delivered (105). Once the intervention is delivered, a proximal outcome (e.g., behavioral response) is observed. It is crucial that the timing of this outcome measurement aligns with the distal goal of the intervention, such as sustained behavior change (107). A meta-analytical review (108) of studies where JITAIs were used concluded efficacy of this solution.

To further enhance efficacy of interventions, a (contextual/causal) MAB can be used to inform decisions in JITAIs, as discussed in (109, 110), while keeping one arm as “no intervention.” By utilizing TS, which addresses exploration—exploitation trade-off, user engagement can be enhanced by targeting habits with exploitation, while also new behaviors would be hopefully promoted via exploration. Moreover, a possible approach to mitigate user burden while sending interventions at various time points involves taking into account the cost of each intervention. This can be achieved by integrating a surrogate reward function into a MAB framework, as suggested by (111). The design of the reward function, which guides the selection of delivered interventions over time, must be tailored to the application, where this algorithm should be used.

### 3.3 Behavioral profiling and lifestyle quantification

To further enhance the behavioral change through personalized interventions, behavioral profiles created from available data can be incorporated (112) and used as a context and to describe user’s internal state. Behavioral profiles are collections of user preferences, objective and subjective information, contextual features, and the statistical and causal relationships between them, both at the individual and population level. These can be further summarized into behavioral patterns describing individual’s current habits. Formed behavioral profiles can then be used not only to inform content of personalized intervention, but also to improve timing, frequency and triggering conditions for interventions, i.e., to inform JITAIs (107). The selection of variables and relationships relevant for behavioral profiling is based on the application’s purpose, available data, and technical, regulatory and ethical restrictions.

Simplest behavioral profiles are explicit profiles, computed directly from the data as a vector of proportions of selected behavior, as proposed in (84). If a relevant information is available in the user’s behavioral profile, timing can be even better personalized towards usual habits. For instance, a suggestion to use the bicycle instead of driving car for commuting to work, sent right after the usual wake up in the morning, could be particularly effective. Conversely, recommending that someone go for a walk at their most likely time, when they would already do so, doesn’t foster the formation of a new habit. Additional value can then be provided, such as suggesting a new, longer, or more challenging route on the way back home to boost physical activity levels.

In order to better understand complex patterns and interactions in the collected data, statistical and causal modeling tools for repeated measurements, as introduced in Section 2.1.3, can be employed. As the data are collected longitudinally, N-of-1 design can be used to understand effects in longitudinal self-tracked data (56) but also to understand individual effect of previously sent interventions. Other machine learning techniques, such as generalized mixed effect regression, tree-based methods, neural networks or XGBoost can also be used for pattern recognition (113). Furthermore, a graphical representation of lifestyle, revealing structure among variables, can be estimated. To learn the structure of a causal graphical representation, causal discovery methods, introduced in Section 2.1.1.1, can be used, as done in a data-driven way e.g., in (114). The effects and bounds are then estimated based on the derived structure. Learning effect from causal graphical structure is particularly useful in cases where intervening directly on the variable associated with the desired behavior is not feasible. Intervening on other variable which has direct causal effect on it, should consequently lead to a change in the desired direction for the target variable (115) as well. Moreover, by utilizing counterfactual framework on learned causal graph can be used for simulating of interventions and their consequences (116).

#### 3.3.1 Health trajectories

Health trajectories can be identified using multi-modal information (117). These can be used not only for tracking disease progression, but also for assessing behavioral changes and informing the size of sliding window in MAB, and evaluating of effects of observed variables in a long-run. The identified personal patterns can then be used for improving personalisation in the interventions and sub-goal hierarchy design. Further, by identifying similar users based on their trajectories, predictions can be utilized to adapt the behavioral change program design to increase chance of effectiveness with growth mixture models (118). In future research, such estimated dynamic patterns could be incorporated to the post-analysis inference by including time-varying effects.

### 3.4 Goals, progress and feedback

The distal goal is divided into an ordered sequence of smaller executable proximal goals, as addressed in Section 1.1.2. A hierarchical set of proximal goals directs the trajectory of incremental daily lifestyle improvements, leading to the establishment of a new habit. In our example to improve physical activity, the proximal goal in SMART formulation can be “*I will walk 7,000 steps at least 5 days in a week for 4 months, to increase my physical activity*.” Based on the currently available data in the system, a progress towards this proximal goal is evaluated by fulfillment at each decision point. The computed progress can be then used as a *feedback* component in the intervention. Feedback contains an objective information encoding either progress towards the goal in form of an absolute or relative value, or an information about fulfilling the goal, strike of achieving goals, or any other option that can enhance motivation. Also comparison to similar people or one’s history could be incorporated (119). The feedback is thus one of the motivational components and should be selected in a personalized way to enhance efficacy of the intervention.

Furthermore, a proximal goal can be regularly re-evaluated, and adapted to be either more challenging or easier. This depends on whether it was fulfilled for a defined number of days in the last period, but also on the target population for behavioral change system, and in clinical settings should always be guided mainly by medical professionals. In any case, proximal goals should be updated and aim to lead to distal goal. When the distal goal is reached, a maintenance behavioral change stage begins.

### 3.5 Motivational and persuasive component

Timing, factual content and type of intervention delivery were already discussed. In case of visual intervention, another design considerations can be made. Especially if the intervention is delivered in a form of text, i.e., via notification or through chatbot, motivational and persuasive components can be incorporated to enhance user’s engagement (120).

**Figure 1:**
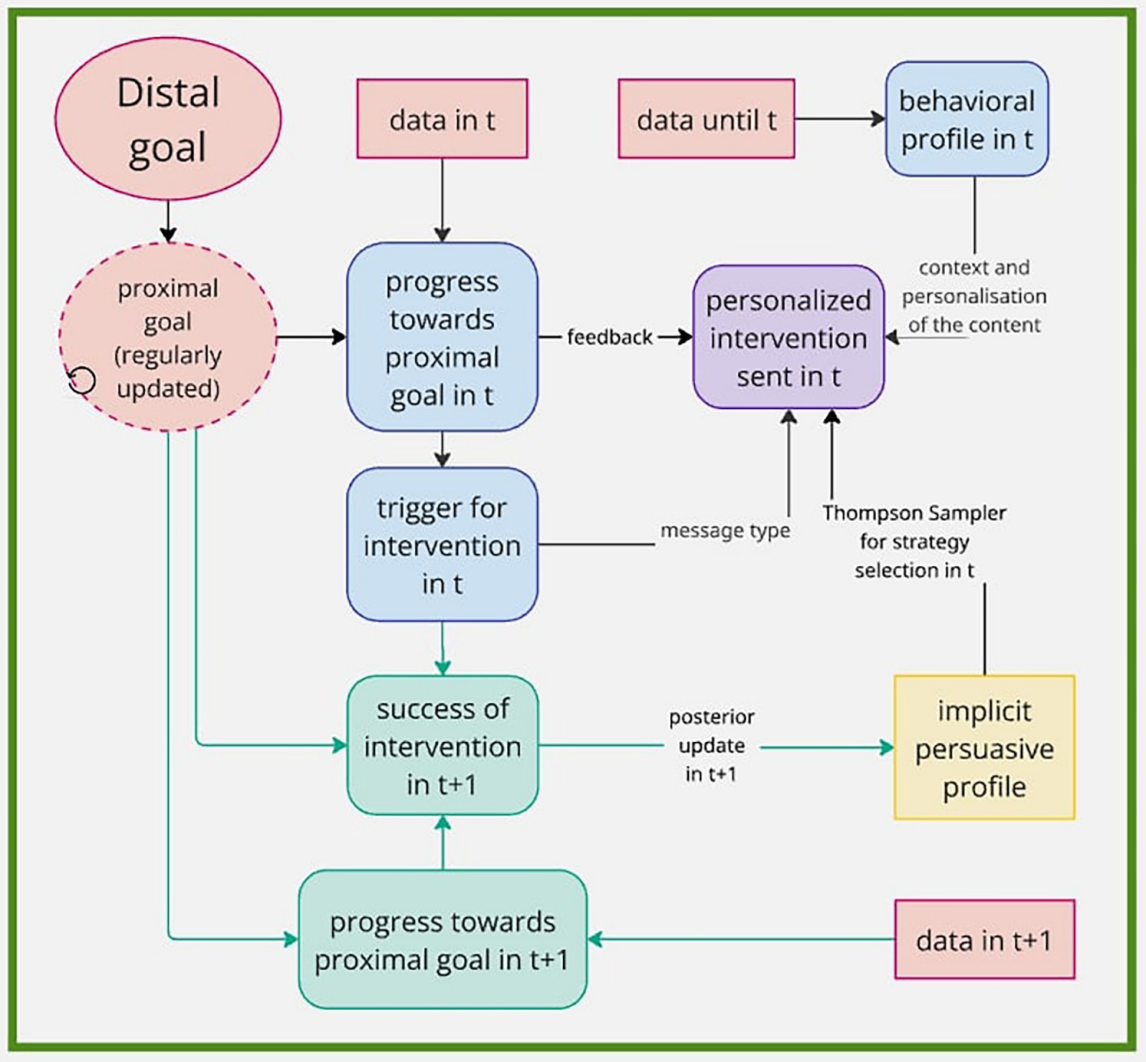
Example of intervention framework using MAB for selection of persuasive strategy in text message. Information from the system are in red, decisions made for intervention creation in time (t) are in blue. Design for update of the implicit profile in time (t+1) is in green. Created using https://miro.com/.

Firstly, the way how the message is presented can be used as a motivational component. In (84) this is facilitated through so-called triggering conditions, which is a set of rules that depend on the evaluated progress towards measurable proximal goal. This can be for example a *praise*, if user already fulfilled the proximal goal, or some type of *suggestion* if the proximal goal was not fulfilled yet. The rules for triggers must be compatible with the aim of the system and should be designed by professionals in the field. Triggering conditions serve not only as motivational component, but also give a base for evaluating whether the user’s behavior is aligned with the aim of the intervention, as described in the example depicted in Figure 1, and can thus help formulate the reward for multi-armed bandit. An example of triggering logic, including triggering conditions and the timing for assessing intervention effectiveness related to our example, is presented in Table 1. Conditions should be always designed based on the available data and target population, and should take into account also contextual state of the user. In special cases with too little available data, this could reduce to a simple scenario with only 1 row.

**Table 1:** Table of example triggers. Based on the condition, given by progress towards proximal goal, the type of the trigger is selected. The proximal outcome is then evaluated at corresponding time.

ID	Type	Condition	Evaluated when	Example
1	Praise	Progress ≥ 100%	End of the next day	Congratulations!
2	Nudge, encouragement	Progress ∈<80%,100%)	End of the day	Almost there!
3	Suggestion	Progress < 80%	End of the day	Take a walk.

The motivational component of the intervention can include real-time and historical observations, whereas a persuasive strategy does not need to rely on the collected data, as it can be solely a persuasive statement. A set of persuasive strategies needs to be defined a-priori. For example Cialdini’s 6 persuasive strategies (121) can be used, as done in (122). A selection of persuasive strategies to enhance adherence to the lifestyle change program can be chosen according to the application purpose and target population. The strategy for the persuasive component, guiding the formulation of the intervention to be sent, can be selected in different ways. A straightforward approach could involve random selection or predetermined fixed probabilities. To allow personalization, a MAB algorithm can be employed. The application of TS for personalized selection of persuasive strategies has been shown to enhance adherence (122), and was also successfully applied to select one of the 6 Cialdini’s principles (123) as a way of *implicit* personalization. This technique is chosen because of its proven efficiency for exploration-exploitation trade-off, and because the posterior distributions recomputed in each step can be viewed as implicit persuasive profiles. Despite the persuasive profiles being only approximate and not correct estimates, they are sufficient for the purpose of the automatic decision-making for interventions (123). For a new user, prior distributions can be either uninformative, or can be jump-started by using prior knowledge. This can be achieved either with relevant questionnaire filled in by the user upon logging in to the system for the first time, as done in (122), or by comparing to similar participants previously enrolled to the study based on the relevant metrics.

Returning to our example related to physical activity, having fixed a daily goal of 7,000 steps at least five times a week, the nudging message could be triggered if the user has taken less than 7,000 steps today and/or has not yet met the step goal in at least five times this week. Furthermore, assuming we know, from contextual/demographic information gathered, that the weather is currently sunny and that the user has a dog, the message may be formulated as follows: *Today you already fulfilled 5,000 steps out of your daily step goal of 7,000 steps. The weather is nice, how about taking your dog for a walk? There are only a few hours left today to fulfill your goal!* This message, depicted in purple in Figure 1, is composed of several components: feedback (*5000 out of 7,000 steps*), contextual information collected automatically from location data (*weather is nice*), contextual information explicitly asked to the user in previous interaction (*take your dog for a walk*) and a persuasive component (*Only a few hours left today*) selected by MAB.

### 3.6 MAB and reward function in mHealth

In the context of mHealth interventions, the integration of MAB algorithms requires careful specification of the reward function. The reward reflects the observed outcome of an intervention and is essential for guiding the posterior update. In adaptive interventions, rewards are typically based on proximal outcomes, which inform the algorithm’s learning and adaptation during the study. However, distal outcomes should also be considered when defining the reward structure, as they ultimately determine the intervention’s effectiveness. Proximal outcomes are evaluated in an online manner based on the triggering condition, i.e., the logic or rule that determines when and why an intervention is delivered. This is illustrated in green in Figure 1. Distal outcomes are assessed during the post-study analysis but should influence the initial reward design to ensure alignment between short-term optimization and long-term goals.

In MAB for mHealth, the reward assigned to an action is determined using data collected after the action is taken. For instance, if a *suggestion* message is sent, the intervention is considered successful if the user performed the desired behavior by the end of the day or up to few hours from the delivery of the intervention. Alternatively, for a *praise* message, success might be defined as the user exhibiting the desired behavior the next day without further prompts. An example of such triggering conditions and their corresponding success criteria are summarized in Table 1. Reward definitions should be tailored to the intervention type, desired behavior change, and data availability. If the reward is binary (e.g., success/failure), a Beta-Bernoulli model is used for the posterior update, otherwise different suitable model must be selected. For further guidance on the design of reward functions in mHealth bandit settings, see (124).

Some considerations for designing of reward function for TS are discussed in (111). Further, examples utilizing MAB in mobile health in various domains involve (77, 109, 125–130). These can also serve as examples for reward design and related considerations. Other examples of RL methods for the application to digital health involve e.g., (131) where authors used Bayesian Mixed Linear Model to approximate the reward, and (132) for multi-agent RL in digital health domain.

### 3.7 Multiple lifestyle pillars

Designing interventions for lifestyle improvements is a complex process, especially if more than one lifestyle pillar should be addressed in a single application. Addressing multiple pillars at the same time could lead to an excessive number of interventions within a short period, potentially burdening the user. Several strategies could be employed to strike a balance between minimizing potential burden and intervening sufficiently to achieve the desired effect. Pillars can be addressed either individually, one-by-one, or simultaneously, with the primary focus on one at a time. As before, automatic selection with MABs could be incorporated to enhance personalization. The design of reward for such decision-making system would be however probably the biggest challenge.

Furthermore, it is worth to consider, that what could lead to the highest reward in short term, might not be the best decision in long-term, as the aim of the system is to change behavior and maintain healthy habits. The reward must be thus designed to reflect both proximal and distal goal at the same time. Moreover, even if interventions would not work as intended at the beginning, they might be effective for behavioral change in the long run. For this, incorporation of full RL with delayed rewards (63) in the later stages of the behavioral change program could enhance overall efficacy.

## 4 Evaluation and inference

To understand the efficacy of the behavioral change system, a comprehensive statistical evaluation is essential. Interventions delivered over longer period of time can be evaluated either via randomized controlled trial (RCT) by assigning participants into control vs. intervention group, or on an individual level, allowing also for evaluation of individual components. In this section, various possible approaches and associated challenges will be discussed, starting from defining the research question.

For proper communication, an estimand must be formulated, providing precise definition of outcomes of the study and other relevant factors (133). For this, a PICO (Population/Problem, Intervention, Comparison, and Outcome) framework, which plays a crucial role in evidence-based medicine (134), can be employed. PICO aims to guide study evaluation, serving to clearly define research questions and inform model specification. In the *population* component, inclusion and exclusion criteria are defined. These have to be inclusive, fair and ethical, but at the same time aiming to answer the research question of interest, while avoiding selection bias. The aim is to enroll a representative sample of the target population. *Intervention* specifies type of intervention delivered, such as tailored digital nudges delivered via a smartphone application, or more generally, usage of the behavioral change system. The choice of *comparator* then defines competing intervention or a standard care. For evaluation of digital interventions this can be no intervention, or not using the system at all, as also only self-monitoring can be an intervention (88). Finally, *outcome* quantifies the research question and guides model selection and analysis. In the example of physical activity this could be daily step count and its change throughout the study. On top of the primary outcome of interest, secondary outcomes can be included, targeting e.g., subgroup-specific effects or time-varying effects reflecting treatment effect heterogeneity over time. To enhance reporting quality of randomized trials, dedicated guidelines should be followed (135).

The study is typically framed as a superiority design aiming to show benefit of intervention against comparator. Digital interventions may however not always outperform existing treatments in absolute effect size, but can still provide meaningful value if they achieve similar outcomes with improved usability or accessibility. For this, a non-inferiority design can be more suitable (136). In the non-inferiority trial, an alternative hypothesis is formulated through a non-inferiority margin. The confidence interval of the treatment effect must then lie outside of the inferiority region defined by the non-inferiority margin (137). The non-inferiority margin, determined in advance, is typically selected based on the results form a previous study, or by an expert, and should depend on the severity of the endpoint (138).

### 4.1 Study design

Study objectives defined by PICO are then defining the quantity of interest. Typically this would be an average treatment effect (ATE), i.e., the causal effect of treatment on the outcome. To evaluate ATE on a population level, an RCT is typically used. RCT is a gold standard for estimating unbiased causal effects, as random assignment facilitates identification of the ATE under minimal assumptions (32, 136).

If the sample size required for a reasonable power of the test can’t be met or ethical considerations do not allow for running an RCT, an observational study designed as one-arm feasibility study (139) can be performed to test preliminary hypotheses of associations and collect feedback. If longitudinal data were collected, an effect of interventions on an individual level can be evaluated, and changes in trends should be visible. Furthermore, potential research designs to evaluate efficacy and effectiveness of mHealth interventions are summarized in (140). The effect of a new treatment on the selected study endpoints collected through one-arm experiment can be then either compared to outcomes in the synthetic control arm (141) or to the accepted conventional value of outcome by a non-inferiority study design (138).

#### 4.1.1 Micro-randomized trials

Post-hoc analysis in RCT provides an evaluation of efficacy of the overall behavioral change program and hypothesis testing, but the information about contribution of intervention components and information for future improvements is limited (142, 143). To understand effectiveness of interventions, a multilevel (fractional) factorial experiment should be employed (143).

**Figure 2:**
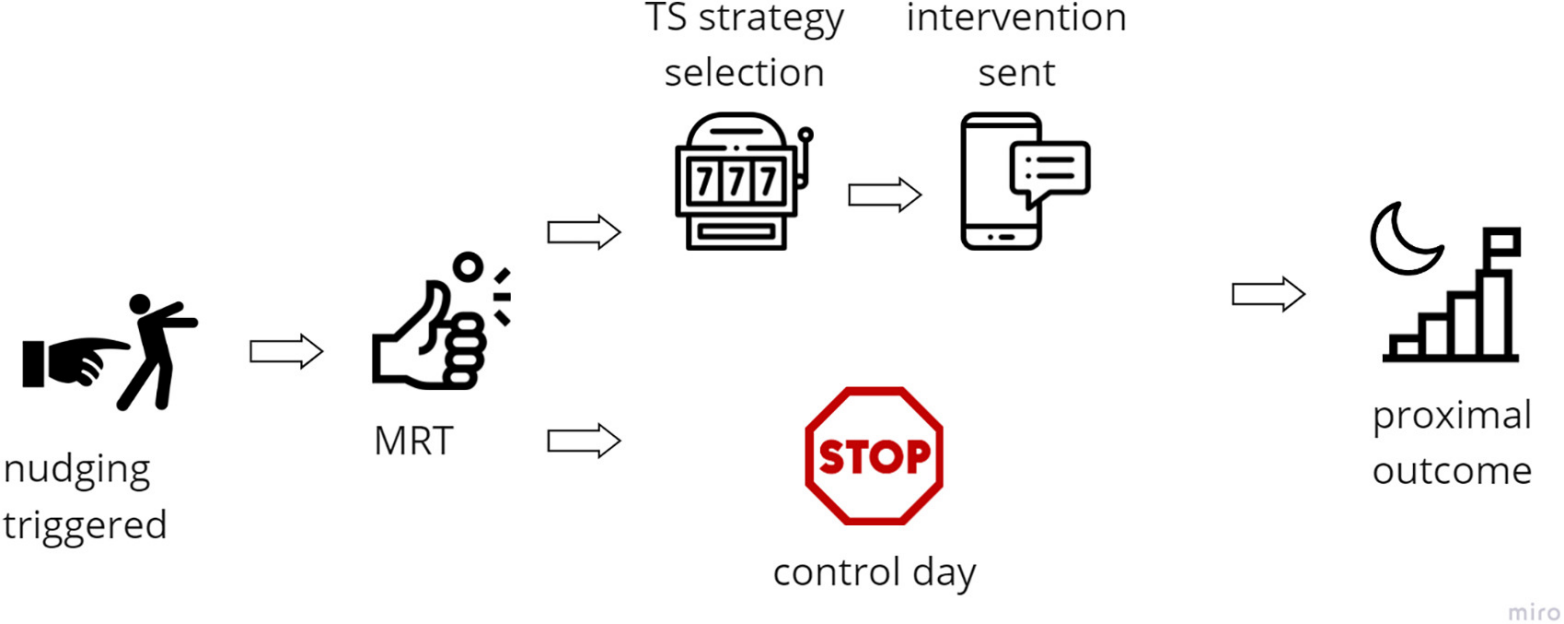
Example of intervention framework using MAB for selection of persuasive strategy with micro-randomized trial. Icons created using https://www.flaticon.com/.

To further evaluate the effectiveness of digital interventions at the individual level and in real-time, *micro-randomized trial (MRT)* offers a powerful experimental design that complements traditional RCT. While RCT assesses the ATE on distal outcomes across a population, MRT focuses on proximal outcomes, i.e., short-term responses to interventions delivered at specific decision points (144). MRT can decrease the sample size compared to a full factorial deign, while still allowing for causal inference of intervention effects (144). In the MRT, participants are repeatedly randomized, often several times per day, to either receive or not receive an intervention at predefined decision points, using fixed probabilities. This repeated randomization allows for the estimation of time-varying and context-dependent causal effects at the individual level (144). An example of MRT design with persuasive component selected by MAB is illustrated in Figure 2.

MRT is particularly well-suited for informing JITAIs, where decisions about intervention delivery incorporate contextual features such as location, mood, or previous engagement (105). MRT supports various outcome types, including binary (145), continuous, and zero-inflated count outcomes (146), and facilitate analysis of time-varying moderator effects (147). MRTs have been successfully applied in domains such as physical activity promotion (125), alcohol reduction through timely notifications (148), and digital cardiac rehabilitation (149, 150). Considerations for sample size for MRTs are discussed in (151).

Although MRTs are designed for short term decision-making and personalization, they do not replace RCTs. Instead, both designs can be used in a complementary manner, for instance assessing proximal effects via MRTs and distal outcomes via RCTs in the same study (150). While MRTs are designed to assess the short-term effects of interventions, understanding their cumulative impact on long-term behavioral or health outcomes requires integrating longitudinal modeling with causal inference frameworks, including those that estimate individual treatment effects over time (99).

#### 4.1.2 Adaptive designs

Traditional RCTs typically use fixed randomization ratios to assign participants to treatment or control groups. However, *response-adaptive randomization (RAR)* improves upon this by modifying allocation probabilities during the trial based on accumulated outcome data. The core idea is to assign more participants to treatment arms showing superior performance, thereby potentially enhancing patient benefit without sacrificing statistical validity (152, 153). RAR is particularly relevant in clinical trials where ethical considerations favor minimizing exposure to inferior treatments. To dynamically select allocations to arms, MAB approach can be used (154), aiming to maximize expected rewards (e.g., engagement, behavioral change). To enhance balance of exploration and exploitation in settings with satisfactory sample size, TS introduced in Section 2.2.3 can be used. For inference from data collected under MAB, incorporating an off-policy learning paradigm (155) could be beneficial. However, further research is needed to fully explore and refine this framework.

The concept of adaptive designs can be applied also to high-frequency interventions such as MRTs, where treatment options are randomized at multiple decision points over time. Instead of using fixed randomization probabilities, data from prior interventions can inform future allocations, optimizing intervention delivery based on individual participant responses. Further adaptation can be achieved through *Sequential Multiple Assignment Randomized Trials* (SMART), where participants are re-randomized based on earlier responses (156). SMART design combines optimization by JITAIs, and utilizes MRT to also allow inference. Moreover, the behavior change program could be then formed by multiple components, focusing on different lifestyle pillars in parallel. The selection of the experimental design must be tied to design of interventions’ components and desired inference; a more detailed overview can be found in (157).

In digital health, these adaptive designs can be embedded within a *Multiphase Optimization Strategy (MOST)*, where interventions are first optimized for individual needs (e.g., through MRT or SMART) and then rigorously tested via RCT to ensure both individual tailoring and robust population-level evidence (142).

### 4.2 Statistical analysis and inference

A clear specification of estimand to evaluate intervention effect is critical for valid statistical analysis, especially in adaptive designs where both exposure and adherence can vary over time. *Intention-to-Treat (ITT)* assesses the effect of the assigned intervention, regardless of whether it was received, preserving the benefits of randomization and minimizing bias (158). On the other hand, *Per-Protocol (PP)* evaluates the effect among participants who fully adhered to the protocol (159). While potentially more relevant for assessing efficacy, this approach risks introducing selection bias due to non-random adherence.

To mitigate this bias, *instrumental variable (IV)* methods can be employed (160, 161). In randomized trials, the random assignment itself serves as a valid instrument to estimate the causal effect of treatment receipt, also known as the complier average causal effect (CACE) (162). In digital interventions, this approach is particularly relevant when user interactions (e.g., notification clicks or app usage logs) can be used as proxies for actual treatment exposure (163).

Given that digital health studies typically involve repeated measurements over time, appropriate longitudinal models are essential. *Mixed-effect models* account for within-subject correlations and allow for estimation of both fixed effects (e.g., treatment) and random effects (e.g., individual-level variation) (164, 165). Depending on the outcome distribution, linear or generalized models should be used. These models can be estimated using methods such as restricted maximum likelihood (REML), the EM algorithm (166), or Bayesian inference (167). It is important to note that fixed effects can only be interpreted marginally if treatment assignment is independent of random effects (168). In addition, *survival analysis* methods such as Cox proportional hazards models can be used to evaluate time-to-event outcomes, such as time to dropout (169). Moreover, several statistical frameworks have been proposed to analyze MRT data, including weighted and centered least-squares estimators (170) and robust inference methods for longitudinal binary outcomes (145). To evaluate individualized treatment effects, facilitating personalized decision-making within the system, machine learning models and concepts from causal inference can be employed (171). Furthermore, *multilevel models* can estimate time-varying treatment effects of proximal interventions delivered repeatedly throughout the study under MRT design (144). Moreover, in the studies with MRTs, although not desired, a carry-over effect might occur and should be controlled for in the analysis. These can involve not only positive effects of interventions from previous days, but also experience of burden.

To ensure the validity of results, especially in the presence of multiple testing and repeated measures, all components of the statistical analysis, including model specifications and hypotheses, should be pre-specified before data collection. This guards against inflated Type I error (172). Furthermore, when evaluating relationships for which randomization was not applied, *causal inference tools*, introduced in Section 2.1.2, can be used to estimate effects from observational data. Approaches such as propensity score adjustment, IPW, or g-methods help reduce confounding. Moreover, findings from both randomized and observational components can be combined using a *causal fusion* framework (51), enabling the integration of multiple evidence sources to strengthen causal conclusions.

## 5 Discussion

This paper reviewed personalized adaptive interventions through the lens of MAB algorithms, focusing on their application in behavioral change contexts. MABs were selected due to their lower initial regret compared to full RL approaches, making them suitable for early-stage interventions where user engagement is fragile. Moreover, experimental designs allowing for proper statistical inference were discussed, ranging from RCT and MRT for individual treatment effect estimation, up to adaptive designs. These methods provide a toolkit for balancing personalization and scientific rigor in digital health. The discussed framework and similar adaptive approaches pave a way for opportunities in personalization of mHealth applications not only for behavioral change, but also for chronic disease prevention and management.

Designing effective and scalable adaptive systems presents several challenges. Interventions must be engaging, ethically sound, and technically feasible. A key difficulty lies in delivering the right support at the right moment. Accurate user profiling can enhance the timing and personalization of interventions, yet further research is needed to fully leverage MABs in this area to maximize adherence and impact without compromising inference. Personalization, while essential for effectiveness, inherently limits generalizability to broader populations. Additionally, behavioral systems must be dynamic, continuously adapting to evolving user profiles and behaviors. This motivates techniques such as sliding windows and non-stationary models to ensure continued relevance and effectiveness. User retention is another critical factor. MABs can help reduce dropout in early stages, where it tends to be highest, by adapting quickly to user responses even with little data.

Despite the promise of goal-setting and motivational strategies, long-term engagement remains a significant challenge. Users may disengage due to a lack of perceived progress, goal misalignment, interface complexity, or poor fit with changing personal circumstances and life contexts (173–176). To address this, interventions should incorporate adaptive feedback loops and re-engagement mechanisms such as timely nudges and dynamic goal adjustments (177). Periodic reassessment of preferences and behavioral stages can help ensure the intervention remains relevant and supportive. While psychological perspectives are increasingly integrated into digital intervention frameworks (23, 28), further empirical research and evaluation across diverse domains of lifestyle change would strengthen the validity of these approaches. Gamification, socialization, education, and engaging feedback mechanisms may also foster deeper involvement. Additionally, fostering intrinsic motivation and self-determination in behaviors might be more effective than relying on extrinsic rewards and external regulation to drive behavior. Moreover, from a psychological standpoint, sustainable behavior change is better facilitated through the pursuit and attainment of learning and process goals, rather than focusing solely on the outcome goals (29). These aspects have to be however carefully translated into the design of AI-enhanced behavioral change systems.

Ethical and regulatory considerations are essential throughout the system’s lifecycle. Interventions must ensure user safety, transparency, and autonomy. Users should retain control over their data, as in EU under the law of General Data Protection Regulation (GDPR, Regulation (EU) 2016/679), and be informed about how decisions are made. Systems must avoid unintended harms, such as delivering inappropriate recommendations or reinforcing harmful behaviors (178). Equity, inclusiveness, and fairness are critical to building trust and ensuring broad societal benefit. Further ethical considerations for usage of automatic decision-making systems in healthcare are summarized in (179). For health-related applications, also regulatory compliance is paramount. Interventions should be developed in consultation with domain experts and follow relevant legal guidelines. In the U.S., the FDA provides regulatory guidance for digital health technologies (180), while the European Union’s Artificial Intelligence Act (EU AI Act, Regulation (EU) 2024/1689) introduces specific requirements for AI-based systems. Notably, behavior change systems, though generally prohibited, are allowed in the medical domain under strict conditions. Certification under the EU Medical Device Regulation (MDR, Regulation (EU) 2017/745) may be required, depending on the intervention’s intended use and decision-making autonomy. For example, automated goal setting in cardiac rehabilitation programs may require oversight from medical professionals to ensure safety. The use of generative AI, such as large language models (LLMs), also raises regulatory challenges. Automatically generated motivational messages may face restrictions, and should ideally rely on professionally vetted content. As discussed in recent literature, the growing role of LLMs in healthcare applications demands further research into appropriate safeguards and governance mechanisms (181). Evaluation of mHealth solutions also requires clear objectives, validated outcome metrics, and longitudinal monitoring. In digital interventions, as in other healthcare applications domains, RCTs are gold standard for evaluation of the system. When dealing with repeated exposures, MRTs offer advantages over traditional RCTs, such as smaller sample size requirements and the ability to assess individual intervention components. This allows for iterative refinement based on real-world data and user feedback, while adhering to the framework of RCT. However, further research is needed to understand generalizability of the results from MRTs. Additionally, the so-called Hawthorne effect (182), where participants alter behavior simply because they are being studied, can inflate perceived intervention efficacy. Therefore, future studies should include post-deployment evaluations in naturalistic settings to gauge real-world effectiveness.

This review highlights a rich and evolving landscape of methods for designing and evaluating adaptive digital health interventions. The main opportunity lies in the real-time personalization offered by MABs and related adaptive methods, which can improve engagement and efficacy while preserving analytical tractability. These approaches enable rapid learning from user behavior and offer fine-grained control over intervention delivery. However, this flexibility also introduces trade-offs. Personalization can reduce generalizability; adaptivity can complicate causal inference; and automated decisions raise ethical and regulatory concerns. While MABs reduce regret in the short term, they may not fully capture long-term outcomes or delayed effects, for which more complex RL methods might be required. Furthermore, deriving causal effects especially from observational data for constructing of behavioral models is a challenge, as it is not possible to derive causal effects purely from observational data without posing (sometimes untestable) assumptions. Current tools for causal discovery offer promising results when the background knowledge is incorporated. However, incorrect direction of edge in the output can introduce incorrect assumptions for the subsequent identification and inference (183). While proper selection of confounders reduces bias in the causal estimates, selection of inappropriate controls, such as colliders or mediators, can result in estimates that are biased even more than uncontrolled estimates (184).

A promising direction for future research is the creation of personalized *digital twins*—virtual representations of individuals built from continuous wearable data and contextual information to capture behavioral trajectories (185, 186). Building of digital twins unifies the key ideas discussed in this review, including MABs, adaptive learning, causal inference, and interventions simulation, within a single, coherent framework. By simulating different intervention scenarios, digital twins can help optimize the timing and content of support while avoiding real-world risks. Integrating causal inference and adaptive algorithms into these models would allow researchers to test and refine intervention strategies virtually before deploying them in practice. This approach could enable highly personalized treatment plans, enhance safety, and accelerate development cycles. However, important challenges remain, such as ensuring model accuracy, managing computational complexity, protecting user privacy, and validating predictions against real-world behaviors. Future work should focus on scalable methods to build and update digital twins in real time, evaluate their advantages over conventional adaptive designs, and develop metrics to measure their precision and impact in digital health interventions.

In conclusion, adaptive mHealth interventions leveraging MAB algorithms, causal inference frameworks, and emerging digital twin technologies represent a promising frontier for personalized behavior change. Their success, however, depends on ongoing adaptation, rigorous evaluation, psychological insight, interdisciplinary collaboration, and compliance with regulatory and ethical standards. Future work should validate long-term efficacy, investigate generalizability across populations, and explore broader applications across behavioral, clinical, and public health domains.
